# Experiences and perceptions of Chinese patients enrolled in a clinical trial assessing tuina and manual therapies for knee osteoarthritis: a nested qualitative study

**DOI:** 10.1186/s12906-025-04926-7

**Published:** 2025-05-28

**Authors:** Luping Liu, Lingyun Zhang, Sina Li, Meiling Cai, Siyu Han, Zhiwen Weng, Qianji Chen, Yixuan Gao, Xiaoming Yang, Yang Zhang, Duoduo Li, Changxin Liu, Ya’nan Sun, Xiyou Wang, Changhe Yu

**Affiliations:** 1 Yongshun Community Health Service Center, Beijing, China; 2 Tuina and Pain Management Department, Dongzhimen Hospital Beijing University of Chinese Medicine, No. 5, Str., Hai Yun Cang, Dongcheng District, Beijing, China; 3 Pediatrics Department, Inner Mongolia Xing’an Meng People’s Hospital, Xing’an Meng, China; 4 Acupuncture and Moxibustion Department, Langfang TCM hospital, Langfang, China; 5 Acupuncture and Moxibustion Department, Luohu District Chinese Hospital, Shenzhen, China; 6 Traditional Chinese Medicine Department, Xuanwu Hospital Capital Medical University, Beijing, China

**Keywords:** Qualitative study, Tuina, Manual physical therapy, Experiences, Perceptions, Knee osteoarthritis, Thematic analysis, Doctor-patient interaction

## Abstract

**Introduction:**

KOA is a prevalent joint disorder significantly impacting patients’ quality of life. Tuina and manual interventions are prioritized in clinical practice within the Chinese healthcare context. Current qualitative studies mostly focus on symptom management and basic disease perceptions, overlooking patient-centered treatment expectations, therapeutic process perceptions, and doctor-patient interaction impacts during manual therapy. This study aims to address these gaps by exploring Chinese KOA patients’ experiences, perceptions, and expectations of manual therapy, emphasizing contextual factors affecting therapeutic outcomes and interactions.

**Methods:**

Participants with KOA were sampled using a simple sampling method from a randomized controlled trial of Tuina treatment versus manual physical therapy (MPT). The interviews were conducted by two researchers who have extensive experience interviewing KOA patients, and data were gathered through face-to-face, semi-structured interviews to ensure a high level of information power. Three experienced researchers subsequently analyzed employing thematic analysis to assess patient experiences and outcomes from both treatment modalities.

**Result:**

The study interviewed a total of 61 participants, thematic saturation was reached when interviewing 42 participants, and seven codes along with 5 sub-themes were utilized to depict potential doctor-patient interactions and influencing factors. This process led to the formation of three themes: Understanding and Impact, Treatment Expectations and Satisfaction, and Treatment Goals and Outcomes, which helped in constructing a model to understand the underlying influences among these themes.

**Conclusion:**

Our study generated three themes—Understanding and Impact, Treatment Expectations and Satisfaction, and Treatment Goals and Outcomes, and developed a manual therapy model based on these themes. The generated model shows the important factors of doctor-patient interaction in KOA manual therapy management. Future research should expand to multidisciplinary and cross-cultural models to align standardized protocols with individualized patient needs.

**Supplementary Information:**

The online version contains supplementary material available at 10.1186/s12906-025-04926-7.

## Strengths and limitations of this study

**Strengths:**

Using a qualitative research design to explore various influencing factors in manual therapy for KOA, we generated a doctor-patient interaction model for manual therapy in KOA, aiming to identify key priorities for clinicians in optimizing treatment strategies and delivering holistic care. Employing a patient-centric approach, capturing diverse patient experiences and perceptions, including potential doctor-patient interactions, for application in medical practice. Providing an in-depth exploration of patient experiences with manual therapy interventions (e.g., Tuina and manual physical therapy), highlighting both perceived benefits (e.g., pain relief) and limitations (e.g., variability in treatment responses).

**Limitations:**

The sociocultural and clinical context of outpatient care in China may limit the transferability of results to populations in other healthcare systems or cultural environments. As a nested qualitative trial, this research inherits limitations from the parent trial, this may affect the flexibility of applying findings to diverse clinical practices.

**Supplementary Information:**

The online version contains supplementary material available at 10.1186/s12906-025-04926-7.

## Introduction

Knee osteoarthritis (KOA), a prevalent and progressive joint disorder, leads to pain, functional limitation, and decreased quality of life in middle-aged and elderly individuals [1–3]. As the population ages and obesity rates rise, its prevalence is increasing, putting greater pressure on both individuals and healthcare systems [4]. Several guidelines highlight that first-line care for KOA should include patient education, weight loss, exercise, and physiotherapy [5–9]. Manual therapy (MT), a key component of physiotherapy, which encompasses various techniques such as Chinese Tuina therapy [10], Thai massage [11], and joint mobilization [12–15], is frequently incorporated into KOA treatment regimens. A recent trial indicates that both Tuina and manual physical therapy can bring about clinically significant improvements in KOA treatment [10].

KOA patients desire a more holistic and personalized treatment approach beyond medication due to the significant impact of the condition on their daily lives [16] and the feeling that their experiences are often minimized by clinicians [17, 18]. Doctors often prioritize other comorbidities and focus on pharmacological treatments, feeling ill-equipped to address lifestyle factors [17, 18]. Patients desire a more holistic and personalized approach beyond medication. This mismatch can result in suboptimal outcomes [19]. Improved communication between patients and practitioners is essential to enhance treatment adherence [17, 18]. Meanwhile, qualitative studies have revealed culturally specific psychosocial issues in Asian KOA patients, which have been identified as important influences on patients’ pain perception and help-seeking behaviors [20].

KOA patients require manual therapy that not only effectively reduces symptoms and improves their quality of life but also meets their expectations for a patient-centered approach with good doctor-patient communication. Although manual therapy is widely used in clinical practice, qualitative research on it remains limited. Research indicates that the effectiveness of manual therapy (MT) in treating musculoskeletal disorders is influenced by patients’ expectations, practitioners’ skills, and doctor-patient communication [21]. Studies show that MT can relax patients, improve their quality of life, and reduce symptoms [22, 23]. When comparing Tuina (a form of traditional Chinese manual therapy) with Western manual physiotherapy, 94% of patients preferred Tuina, which they perceived as gentler [24]. Additionally, MT’s patient-centered approach enhances doctor-patient communication [25], which can positively influence patients’ expectations of the treatment [26]. However, there is still limited research exploring KOA patients’ experiences, expectations, and perceptions of MT, particularly within the Chinese cultural context. Addressing this gap is crucial for aligning clinical practices with patient needs and improving holistic care strategies.

Therefore, our study aims to uncover patients’ experiences and perceptions regarding KOA and manual therapy, to explore influencing factors and feedback unique to doctor-patient interactions in the Chinese context and their possible influence on the entire treatment journey, and investigate key reference points for integrating patient feedback in the development of manual intervention strategies by Chinese clinicians, ultimately optimizing clinical decision-making efficiency.

## Methods

### Study design and sample

This study is a qualitative sub-study of a randomized controlled trial (RCT). The purpose of the trial is to evaluate the effectiveness and safety of Tuina treatment for KOA compared to manual physical therapy (MPT). A total of 140 KOA patients who ranged in age from 40 to 75 years were included. The participants were randomized to receive either Tuina or MPT. The trial is available on the NIH Clinical Trials website (registry ID: NCT03966248,2019-05-26) and the results of the study were published separately [10].

Preliminary findings from the host trial indicated that Tuina produced beneficial effectiveness similar to MPT in treating KOA. The qualitative study is a sub-study of the trial to explore patients’ perceptions of KOA and of the treatment modalities. An inductive logic study design [26–29] was used for this qualitative study, following established qualitative research methodologies. The main criterion for determining saturation was whether the new data could contribute to the production of new theoretical insights [30, 31]. Although no specific minimum sample size was predetermined, qualitative research typically seeks a diverse and rich sample. A sample of 30 participants is generally considered sufficient for information saturation in qualitative studies [32]. However, recent qualitative research discussions advocate for an information power model instead of a saturation-based approach. This model takes into account factors like study aim, sample specificity, theoretical application, analytical strategy, and dialogue quality [30]. We therefore adopted this method to ensure our findings were information-rich. In brief, information power posits that the amount and quality of information a sample provides determine the required number of participants [30]. The interviews were conducted by HSY and CML, who have extensive experience interviewing KOA patients about their experiences, perceptions, and expectations. Their expertise and deep understanding of the topic enabled focused and high-quality dialogues. We recruited 140 trial participants via simple sampling using robust inclusion and exclusion criteria, and 61 responded. Using these samples, we employed an exploratory, inductive thematic analysis to assess KOA patients with diverse characteristics. Although no specific theory guided the cross-case analysis, three experienced researchers conducted the analysis to achieve analyst triangulation and developed the dictionary prior to formal interviews and analyses, fully exploring variations and ensuring comprehensive data understanding.

### Strategy of inquiry

The informed consent for the interview was conducted simultaneously with the inclusion of the main study by signing the participant’s informed consent form. The consent was confirmed verbally again at the beginning of each interview. The semi-structured interviews [33, 34] were conducted by one-on-one, face-to-face at baseline, after the first treatment, and at the end of treatment. The interview site was a separate quiet clinic room, and the interviewer introduced himself or herself before the interview, explained the purpose, methods, significance, and time requirements of the study to the participant, promised to follow the principles of confidentiality and anonymity.

The interview lasted 15–20 min, using an interview guide (Table 1). The interview guide includes the experiences and perceptions of participants about the manual therapy and KOA before the treatment, after the first treatment and in the end of 4 weeks of treatment, as well as the perceived effects (including but not limited to physiological effects) and changes in health and wellbeing (Table 1). The interviews were conducted as semi-structured sessions by two researchers (HSY, CML) who did not participate in the clinical intervention. The participant was guided to investigate and answer some of the questions in depth, then the interview was completed when it was determined that the information in the listed interview outline was saturated.

**Table 1:** Interview guide

Questions	Timepoints
1. Have you ever received any treatments in the past to address your condition? (For example, have you tried manual therapy?)	1,2,3
2. Do you, or do any of your friends or acquaintances, have any familiarity with manual therapy? If so, could you kindly share how you or they learned about it?	1
3. What are your thoughts regarding whether manual therapy could be beneficial for your specific issue? If you believe it could help, could you kindly express your expectations regarding how much it might help you?	1
4. Could you please describe the symptoms related to your knee discomfort, including when you first noticed them and the most noticeable signs?	1,2,3
5. Has your knee condition had an impact on your day-to-day life?	1,2,3
6. How well-informed are you about your condition, including your level of understanding and the sources of information you’ve relied upon?	1
7. Could you provide a general overview of your experiences with manual therapy, including any physical sensations or emotional responses?	1,2,3
8. How would you describe the manual therapist’s technique and your sensations during the treatment, including any communication or rapport you may have had with the therapist?	2,3
9.Following your treatment sessions, how would you rate your level of satisfaction? If you’re comfortable, please explain what factors contributed to your satisfaction or dissatisfaction, and share your perceptions of the treatment’s effectiveness (improvement and perceived efficacy).	2,3

Timeponit1: baseline; Timepoint 2: after the first treatment, Timepoint 3: the end of treatment

The interviews were recorded by audio recording equipment and transcribed by one researcher (HSY) into NVivo software. When the transcribed data were in doubt, the data were checked by a return survey of the participant, and the transcription results were reviewed by another researcher (CML).

### Data analysis

Thematic analysis was used to analyze the interview results. The thematic analysis consisted of six steps, including familiarizing with the data, generating initial codes, searching for themes, reviewing potential themes, defining and naming themes, and generating reports [35].

Data analysis was carried out by three experienced manual therapy researchers (LLP, ZLY, and HSY) to achieve analyst triangulation. After independent coding, all analysts convened in regular reflexive meeting to discuss positional assumptions, compare code assignments, resolve discrepancies, and refine the coding framework. Throughout these discussions, we critically examined how our clinical backgrounds in manual therapy might influence data interpretation, ensuring that themes were grounded in participant accounts rather than researcher preconceptions.

Three researchers (LLP, ZLY and HSY) who maintained no prior engagement with the trial’s therapeutic procedures independently coded three pilot transcripts manually, creating a codebook. They employed continual comparison methods, using memos and inquiries to define category boundaries, assign data segments systematically, and summarize each category’s content, ensuring that categories accurately represented the data rather than constraining data to predefined categories and those with poor coherence were communicated and questioned to a senior researcher (YCH) and determined by discussion to further enhance the credibility of the findings. NVivo software was used to assist the analysis. The initial codes were analyzed into potential themes and to condense the thematic sections. [36]. Repeated corrections were made to ensure the essence and meaning of each theme, and then sub-themes of appropriate meaning were identified. The final step was to integrate the interrelated themes into the domain.

Coding/theme saturation was determined by the presence of new codes or themes. A comparative method [37]was used to determine thematic saturation. The results of the analysis of each new round of interview data were compared with the results of the previous data analysis to clarify the number of new and repeated themes obtained in the most recent interview, and data saturation was considered to be reached when no more new codes/topics emerged.

### Ethics statement

The study protocol, consent form, and all recruitment materials were approved by the Dongzhimen Hospital affiliated to Beijing University of Chinese Medicine (Beijing, China). The main clinical trial was registered at ClinicalTrials.gov (NCT03966248,2019-05-26).

## Result

### Participants

140 participants were enrolled in the main clinical trial at baseline. All were invited to join the sub-qualitative study, and 61 participated the interview, while 5 patients did not complete all three interviews due to busy work or personal preference. In the process of data collection and analysis, thematic saturation was reached when interviewing 42 participants. And additional 19 participants were also completely analyzed to thoroughly explore potential variations and ensure a comprehensive understanding of the data, yet no new themes emerged.

Table 2 show the characteristics of the 61 participants and those who did not join the qualitative study. The average ages of the 61 participants were 60.61 ± 7.62 years, and most people were Han nationality (57/61, 93.4%) and female (48/61, 78.7%).

**Table 2:** The characters of included 61 participants and those who did not participate in the qualitative study

Variable		Frequency or Mean	
		Participants of interviews (*n* = 61)	Others (*n* = 79)
Race			
	Han	57(93.4%)	75 (94.9%)
Gender			
	Female	48(78.7%)	61(77.2%)
Age, y, mean ± SD		60.61 ± 7.619	59.85 ± 8.251
BMI, kg/m^2^, mean ± SD		25.12 ± 3.370	24.97 ± 2.645
Knee			
	Left	35(57.4%)	32(40.5%)
K-L Grade			
	2	39(63.9%)	37(46.8%)
	3	22(36.1%)	42(53.2%)

A total of 3 Themes, 5 Sub-Themes, and 7 Codes were formed and outlined in Table 3 and described below.

**Table 3:** Sub-themes, themes and domains identified in the qualitative study

Theme	Sub-Theme	Code
Understanding and Impact	KOA Information Gap	Patients’ limited understanding of KOA due to inadequate access to information.
		Improper exercise as a common trigger for KOA
	Impact on Daily Life	KOA significantly impacting daily activities, exercise routines, and overall life enthusiasm.
Treatment Expectations and Satisfaction	Prior Treatment Influence	Patients’ prior treatment experiences influencing their expectations for subsequent treatment.
	Doctor-Patient Interaction	Factors affecting patient satisfaction, including the doctor’s approach, attitude, and patient-physician interaction. Positive relationships and emotion enhance outcomes
	Procedural Concerns	Patient concerns about the intensity and timing of procedures
Treatment Goals and Outcomes	Goals and outcommes	Primary treatment goals include pain symptom relief and restoring athletic ability

**Fig. 1:**
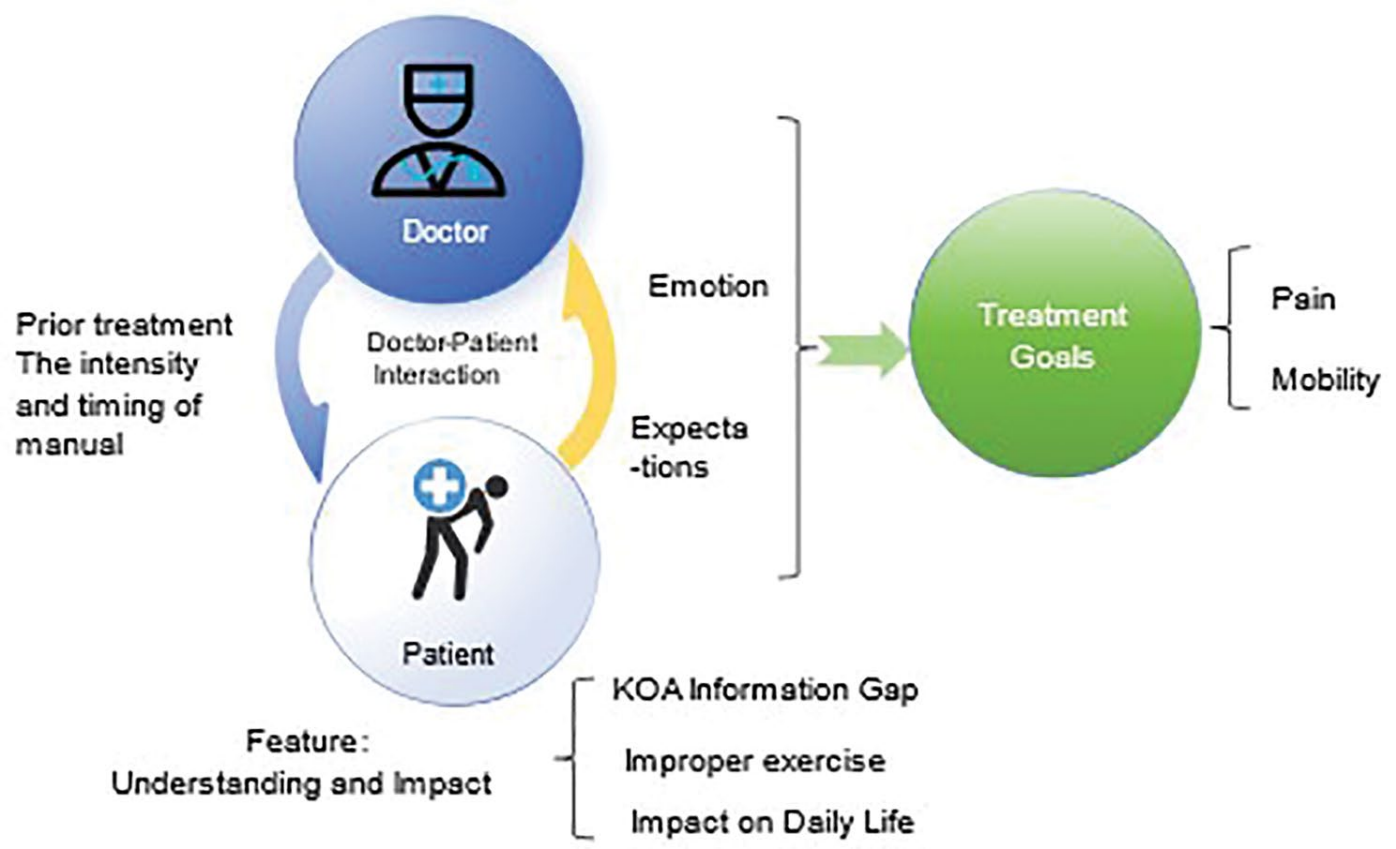
Potential relationships among the synthesized themes

Figure 1** Potential relationships among the synthesized themes** presents a culturally grounded model of doctor-patient interactions in knee osteoarthritis (KOA) management in China, synthesized from thematic analysis of qualitative interviews. This model highlights the dynamic interplay of socio-cultural, clinical, and interpersonal factors shaping treatment experiences and expectations. The model is detailed below: Patients with KOA generally experience the “Information Gap” and the multiple impacts resulting from KOA. Within the **Central Feedback Loop** of the doctor-patient relationship, encompassing “Prior Treatment”, “manual techniques”, “Emotions” and “Expectations”, both parties collaboratively work towards achieving “Treatment Goals” centered around improving patient “Pain” and “Activity”.

### Central feedback loop

Patients’ past interactions with healthcare and outcomes form the initial expectations and emotions when interacting with doctors. Additionally, perceptions of treatment characteristics, such as duration and intensity, can further reinforce these expectations positively or negatively. The bidirectional arrows between Doctor and Patient signify iterative adjustments in communication and treatment plans.

### Interpretation

#### Theme 1: Understanding and impact

##### Sub-theme 1: information gap

The discovery of the “Information Gap” theme underscores a noteworthy aspect of patients’ experiences with KOA. It reveals that many individuals, including 25 participants, grappling with KOA may not possess a comprehensive understanding of their condition. This gap in knowledge is often rooted in the challenges patients encounter when seeking accessible and understandable information about KOA.

This theme shines a light on the complexities of patient education within the healthcare system. It suggests that despite receiving a KOA diagnosis, some patients may remain uncertain about the nature of their condition, its progression, and the available management options. This uncertainty can be attributed, in part, to difficulties in accessing reliable and patient-friendly resources. The following dialogue comes from several different participants. Although they may have heard of or know about KOA, their understanding often remains at a superficial level, such as merely recognizing it as a disease.

Patient53(gender: female, age: 58, Kellgren-Lawrence grade:III): Osteoarthritis, I’ve already had one treatment, so I’m not sure if I know about it, but I’m confused anyway.

P24(female, 45, K-L grade:II): I don’t know, I just read a lot of books about degenerative diseases in old age.

P5(female, 73, K-L grade:III): Well, I don’t have any understanding of it. I just know it’s not life-threatening, that’s it.

P11(female, 53, K-L grade:III): Not quite, I just keep an eye on how to make it (knee) feel better.

It is evident that patients’ lack of proactive engagement with their health condition, such as KOA can be influenced by multiple factors, including limited access to health education and the impact of their living environment. These factors may deter patients from actively seeking information about their condition, thereby contributing to the Information Gap.

P8(female, 74, K-L grade:III): Well, ever since I had this condition more than ten years ago, I attended various seminars in different hospitals to learn about it.

P41(female, 66, K-L grade:II): It’s just that the doctor explained it to me, it’s a degenerative condition.

P2(female, 63, K-L grade:III): I’ve gained some understanding from online information.

P36(male, 68, K-L grade:III): The other day, I watched a video from Dongzhimen Traditional Chinese Medicine Hospital, with the director from the Health Preservation Hall. I recognized some of it.

On the other hand, the “Information Gap” also reflects, to some extent, the misperceptions regarding the causes of the disease and the appropriate treatment methods, particularly in the selection of exercise. Many KOA patients may engage in improper or excessive exercise without considering the specific needs of their condition, potentially worsening symptoms. The diversity in the exercise choices made by patients, which may not always be suitable or properly guided, can contribute to the progression of the condition and an increase in discomfort.

P52(female, 51, K-L grade:II): There were a few of us, and we went to the garden with our dance team. No one was supervising, so we started dancing. I was dancing, dancing, dancing, and then my knee swelled up. It felt really uncomfortable, you know, like a cool sensation. I got a bit scared and couldn’t dance much after that. I had to use some patches for treatment, but whenever I moved, it hurt. Dancing became quite challenging.

P20(female, 59, K-L grade:III): Many years ago, when I was young, I used to be an athlete, probably around 78 years ago. I used to work out at the gym quite intensively.

P41(female, 66, K-L grade:II): About three years ago, when I was climbing Huangshan Mountain, I think I may have injured my knee joints severely. The mountain had steep slopes, and it was quite high, especially the backside. Climbing the front side didn’t cause any problems, but when I climbed the backside, that’s when the issues started. Since then, I began experiencing knee pain.

P60(female, 52, K-L grade:II): The pain would typically occur after I exercised. I’m quite active, especially in badminton.

P25(female, 70, K-L grade:II): Well, I’m not sure. It started with a mild ache, and I didn’t pay much attention to it at first. But over time, it got worse. Now, I have to make sure my legs don’t get too cold because that aggravates the pain.

##### Sub-theme 2: impact on daily life

The sub-theme “Impact on Daily Life” underscores the profound ramifications of KOA on individuals’ everyday existence. It reveals how KOA transcends being a mere medical condition and infiltrates nearly every facet of one’s life. This interpretation elucidates several key aspects:

Quality of Life: KOA significantly diminishes an individual’s overall quality of life. The persistent pain and physical limitations impede one’s ability to engage in routine activities, resulting in a diminished sense of well-being.

P23(male, 69, K-L grade:III): I can’t go on outings anymore, like going to the park for a leisurely walk. If I walk for a bit longer, it starts hurting.

Functional Limitations: Individuals with KOA experience a notable decline in their functional capabilities. Simple tasks such as walking, climbing stairs, or even standing become arduous challenges. This limitation can lead to a loss of independence and a reliance on others for assistance.

P28(male, 74, K-L grade:III): Primarily, what I wanted to do, like playing badminton and other sports, has decreased. Even activities such as shopping can lead to lower back pain and leg discomfort.

Psychological Impact: The constant pain and reduced mobility can have a profound psychological impact. Individuals may experience frustration, anxiety, and depression due to their limited ability to partake in activities they once enjoyed. This emotional burden further exacerbates the overall impact on daily life.

P34(female, 67, K-L grade:III): When the condition flares up, it becomes extremely troublesome. I can’t even walk; my legs need to stay straight. Getting out of bed is difficult, and even turning over is a challenge.

Social Isolation: KOA often results in social isolation as individuals may withdraw from social gatherings and activities due to the pain and discomfort associated with mobility. This isolation can lead to a sense of loneliness and detachment from social circles.

P41(female, 66, K-L grade:II): Certainly, going to work has become somewhat inconvenient. My legs hurt, but I still need to do things like house renovations and buying materials. With no one else available, I have to do it myself.

P15(female, 62, K-L grade:III): Ugh, this is hitting me so hard. I can’t even play badminton anymore, and now it feels like I can’t even take photos either. What am I supposed to do next? Dancing’s out of the question too—it’s just too much. Plus, the friends I used to hang out with haven’t talked to me in ages. Honestly, everything sucks right now.

Social Roles: The condition can also disrupt one’s roles and responsibilities within the family and community. Individuals who were once active contributors may find themselves unable to fulfill their previous roles, causing strain on relationships and personal identity.

P38(female, 51, K-L grade:II): When the pain flares up, I can’t walk anymore. It’s difficult to go up and down the stairs. Sometimes, when I go to buy groceries at the entrance of the community, I have to take breaks along the way.

P47(male, 60, K-L grade:II): I was terrified when I couldn’t get up from the squat toilet. I was so scared, thinking I might never get up. I remembered there was a handle in the toilet and pulled it two or three times, which helped me get up. Even so, I’m still scared.

Coping Strategies: To cope with the impact of KOA on daily life, individuals may develop adaptive strategies. These strategies could include lifestyle modifications, the use of assistive devices, or seeking alternative therapies, such as massage or physical treatments.

P34(female, 67, K-L grade:III): I’ve to undergo treatments for KOA in the hospitals in Beijing and Luoyang cities every year… Later, I also receive treatment in a traditional Chinese medicine hospital in the Zhejiang City.

#### Theme 2: treatment expectations and satisfaction

##### Sub-theme 1: prior treatment influence

Patients’ past treatment experiences significantly shape their expectations and satisfaction with KOA management. Positive experiences foster trust and optimism, while disappointments may lead to skepticism and emotional impact. The same treatment can lead to different outcomes and expectation for different people. Understanding this influence is crucial for tailored care.

P15(female, 62, K-L grade:III): I was extremely dissatisfied with it(acupuncture). I didn’t feel anything at all, no sensation whatsoever. Because I was desperate, that’s why I came to your clinic to give it a try.

P49(female, 60, K-L grade:II): I didn’t consume anything at first, and then I started taking glucosamine for my knee. I took it for a period of time, but I didn’t experience any significant improvement.

P26(female, 62, K-L grade:II): I went to Hospital for some medical check-ups. Then they gave me three shots of hyaluronic acid, one a week, but it didn’t work.

Some patients have experienced positive treatment outcomes and improved quality of life, thereby gaining renewed confidence in future therapeutic interventions.

P27(female, 61, K-L grade:II): After the acupuncture on my knee, it still hurts, but not as much as before. Just occasionally, it can be really painful. Still, I’m quite willing to continue with traditional Chinese conservative treatment.

P34(female, 67, K-L grade:III): Take my walking for example—it’s gotten a lot better. Before, I couldn’t even take a step without struggling. Now, I can manage pretty well.

##### Sub-theme 2: Doctor-patient interaction

Doctor-patient interaction in the context of KOA extends beyond clinical matters to encompass emotional values. The quality of the relationship and the emotional support provided by healthcare professionals play a pivotal role. Trust, empathy, and effective communication are vital in addressing patients’ emotional needs. A positive emotional connection between doctors and patients can significantly impact satisfaction and the overall treatment experience. Patients often seek not only medical expertise but also emotional reassurance and support from their healthcare providers, making the emotional dimension a crucial component of doctor-patient interaction in KOA management.

P5(female, 73, K-L grade:III): Yesterday afternoon, we went to see Dr. Sun for a massage. We had scheduled our appointment for Monday, Wednesday, and Friday afternoons, and it happened to be one of the evenings when he had a night shift. We arrived around 3 o’clock in the afternoon. Dr. Sun is a very handsome young man. He has a great temperament, excellent medical skills, and a very friendly attitude. He is very caring towards the elderly, and he provided me with an excellent massage.

P46(female, 70, K-L grade:II): He (practitioner) pressed right on the spot where my leg hurts, and it was pretty painful. He told me not to complain, but I said it hurt, haha. He said some pain was normal and it would lead to better results. It was my first time, but the doctor was patient in explaining, so I trusted him. I could tell it was working. Originally, I thought that after the first session, the treated area would hurt. But the next day, when I woke up and moved my leg, it felt much more relaxed.

##### Sub-theme 3: procedural concerns

Within the context of KOA management, patients often express concerns regarding the nature of hands-on techniques and the intensity and timing of procedures. They seek assurance that these treatments align with their comfort levels and expectations, especially considering that these techniques are non-invasive and focused on promoting relaxation and relief.

P15(female, 62, K-L grade:III): The treatment effect is quite good, it’s good, but it seems like your treatments are relatively short in duration.

P20(female, 59, K-L grade:III): For me, perhaps the technique used is relatively gentle because, in my case, I’ve had multiple sessions, and I can tolerate it quite well.

P6(female, 67, K-L grade:III): I feel that it’s a bit too gentle because my legs are quite severe. I think the technique could be a bit firmer. Can it be increased a bit? I’ll come again on Saturday.

P26(female, 62, K-L grade:II): The doctor’s technique was great, a nice balance of firmness and gentleness. He wasn’t too light, but even when he applied pressure, it didn’t hurt. He tried to flatten my leg but saw a gap underneath and said it wouldn’t lie flat. He suggested elevating it while sleeping or using a pad. After the session, my leg felt warm and much more relaxed. Really, it felt so much better. On the way back by car, though, it felt a bit cool.

#### **Theme 3** treatment goals and outcomes

The overarching treatment objectives for KOA management primarily revolve around two key facets: alleviating pain symptoms and restoring patients’ athletic capabilities. These goals signify the core focus of therapeutic interventions in KOA care. Pain relief aims to enhance patients’ quality of life and mitigate the discomfort associated with the condition. Simultaneously, the aspiration to restore athletic ability underscores the importance of helping patients regain their physical function and mobility, ultimately fostering a better overall well-being. These primary treatment goals not only guide clinical decisions but also reflect patients’ aspirations for a more active and pain-free lifestyle in their KOA journey.

P41(female, 66, K-L grade:II): Of course, I mean as long as climbing stairs doesn’t hurt.

P35(female, 70, K-L grade:II): I hope that I won’t experience this occasional pain anymore because it does affect my daily life. For instance, at work, when I go to the restroom, unlike at home where there’s a toilet, I squat for a short while, maybe just two minutes, and when I stand up, it becomes uncomfortable. Also, when I have to go up and down stairs, like when driving in heavy traffic or taking the subway, sometimes I have to climb stairs. I move very slowly, not only due to a slow pace, but I also feel like I’m blocking others. I have to take it step by step. In the past, when I had no issues, I didn’t mind taking the stairs or escalators. I preferred to walk, even if there was an elevator or escalator available. But now, if I’m running late, I’ll wait for the elevator because I’m afraid of causing further harm.

P36(female, 68, K-L grade:III): Of course, getting cured is the best, but if the symptoms could be eased, I’d be satisfied too.

These statements reveal a common expectation that treatment should alleviate or even eliminate pain. While many therapies can reduce pain, complete elimination is difficult, especially in progressive diseases like KOA. Moreover, due to the subjective nature of pain perception—some patients tolerate mild pain, while others see slight discomfort as a major burden.

Beyond pain relief, many patients express the desire to regain their previous levels of physical activity. Patient expectations for functional recovery may be influenced by age factors. Younger patients tend to aspire to return to high-intensity physical activities.

P13(female, 45, K-L grade:III): I just want to be able to do sports normally.

P33(male, 46, K-L grade:II): I used to love playing badminton, and I really hope that after treatment, I can finally get back on the court and play with my buddies again.

In contrast, older patients are more concerned with ease of daily activities and maintaining independence.

P10(female, 61, K-L grade:III): I hope I can stand up easily. I don’t want to struggle when climbing stairs. I wonder if I should lead with this leg and then follow with the other.

P47(male, 60, K-L grade:II): I just don’t want to get stuck squatting down anymore, you know? A little pain is fine—I can handle that—but not being able to get up again. That’s what scares me.

## Discussion

The three principal themes—“Understanding and Impact,” “Treatment Expectations and Satisfaction,” and “Treatment Goals and Outcomes”—provide a comprehensive view of KOA patients’ experiences and perceptions, highlighting unique aspects of their participation in manual treatment clinical trials. Figure 1 presents a grounded model of doctor-patient interactions in KOA manual therapy in China, synthesizing these themes into a cohesive framework.

This model illustrates how KOA patients commonly experience an “Information Gap” and the multifaceted impacts of the disease. Within the interactive doctor-patient feedback loop, key elements such as “Prior Treatment”, “Manual Techniques”, “Emotions”, and “Expectations” dynamically influence treatment experiences. These factors shape both patients’ and physicians’ perspectives, ultimately guiding the formation of shared “Treatment Goals”, which focus on alleviating “Pain” and improving “Activity”.

Furthermore, the bidirectional arrows in Fig. 1 emphasize the iterative nature of communication and treatment adjustments, reflecting the dynamic and collaborative aspects of KOA therapy in China. Patients’ previous healthcare experiences and treatment outcomes influence their expectations and emotional responses, while their perceptions of treatment duration, intensity, and effectiveness further reinforce these expectations. Physicians, in turn, adapt treatment strategies based on ongoing patient feedback, ensuring a personalized approach.

This interactive model underscores the complexity and mutual engagement inherent in KOA manual therapy in China. It highlights the need for patient-centered care, where tailored communication, continuous reassessment, and shared decision-making contribute to optimized treatment experiences and outcomes.

Gillian Hawker’s [38] delineated factors across four levels—community, healthcare system, healthcare provider, and client—in their multilevel frameworks. Both this framework and the Chinese culturally grounded model for KOA management prioritize pain relief and functional improvement (e.g., “Activity” in the Chinese model vs. “exercise adherence” in Hawker’s work) as central treatment goals, while acknowledging patient expectations and emotional responses as critical drivers of engagement. The Chinese model further emphasizes the role of “Prior Treatment” in shaping patient expectations, aligning with Hawker’s findings on how physician messaging and delayed referrals influence patient beliefs and adherence. However, the Chinese framework explicitly integrates socio-cultural factors (e.g., hierarchical doctor-patient relationships, traditional medicine influences) and addresses the “Information Gap” (e.g., limited health literacy), themes absent in Hawker’s framework, which instead focuses on structural barriers such as Canada’s public-private healthcare system and financial access to physical therapy services. Additionally, the Chinese model features a Central Feedback Loop that dynamically integrates emotions, expectations, and treatment adjustments to emphasize relational dynamics, whereas Hawker’s framework identifies unidirectional barriers (e.g., healthcare system delays) that disrupt care continuity without an explicit feedback mechanism. These distinctions underscore the interplay of cultural context and systemic structures in shaping KOA management approaches.

### Understanding and impact

Within the overarching theme of “Understanding and Impact,” the two sub-themes—Information Gap and Impact on Daily Life—provide insights into common characteristics among KOA patients and the potential interplay among these aspects. These sub-themes are not entirely isolated, and the Information Gap, to some extent, contributes to the broader impact, especially through triggers like improper exercise.

The reasons behind patients experiencing a KOA information gap are diverse, with two main contributing factors: a lack of motivation to seek information and limited access to information sources [19, 39]. For qualitative researchers, this theme invites exploration into the personal stories and experiences of individuals who have faced these challenges. It opens the door to understanding the emotional impact of grappling with a condition while feeling inadequately informed. Through qualitative inquiry, we can delve deeper into patients’ perspectives, shedding light on the specific barriers they encounter when seeking KOA-related information.

While similar challenges exist globally, the extent of information asymmetry in China is exacerbated by cultural and systemic factors within the healthcare system. Unlike decision-making (SDM) which is increasingly emphasized, China’s traditional medical culture positions physicians in a dominant role within doctor-patient interactions. Patients tend to adopt a passive role, relying heavily on doctors’ decisions and rarely questioning medical advice. This hierarchical structure often limits opportunities for patients to engage in meaningful discussions about their treatment options, thereby widening the information gap.

The exercise section emphasizes the dual role of exercise in KOA [40, 41], where it is recommended as a treatment measure but can also act as a trigger for discomfort if not performed correctly. It highlights a common characteristic among patients—the paradoxical relationship they have with exercise. It also implies that the Information Gap identified earlier might contribute to patients engaging in improper exercise practices, serving as a representative trigger for the condition. This paradox underscores the complexity of managing KOA [42]. This misunderstanding often stems from inconsistent or insufficient medical advice, as well as the variability in information obtained from different sources such as healthcare providers, online platforms, and personal networks. The complexity of exercise selection in KOA management lies in balancing joint protection with maintaining mobility and function. Patients require personalized exercise plans tailored to their condition, yet the lack of standardized guidance and patient education often leads to confusion and suboptimal treatment outcomes. Addressing this issue requires a more structured approach to patient education, emphasizing the importance of appropriate exercise regimens, such as low-impact activities that strengthen muscles around the knee joint without causing excessive stress. Furthermore, enhanced communication between doctors and patients is essential to dispel misconceptions and foster adherence to evidence-based rehabilitation strategies. By narrowing the “Information Gap” in this area, KOA management can become more effective, ultimately improving patient outcomes and quality of life. KOA significantly impacts daily activities, exercise routines, and overall life enthusiasm [43]. Impact on Daily Life sub-theme underscores the substantial burden that KOA places on patients’ quality of life. Simple tasks like walking, climbing stairs, or standing become challenging, affecting patients both physically and emotionally.

### Treatment expectations and satisfaction

Within the “Treatment Expectations and Satisfaction” theme, three sub-themes—Prior Treatment Influence, Doctor-Patient Interaction, and Procedural Concerns—reveal crucial dimensions of KOA management.

Patients experiencing significant limitations in their daily activities due to KOA tend to have higher expectations from treatments. The more severe the impact on their lives, the greater the anticipation for relief and improved functionality. Additionally, prior treatment experiences shape these expectations, with positive experiences fostering optimism and negative ones leading to skepticism. Effective treatment not only mitigates the impact of KOA but also positively influences patients’ expectations.

The intricate connection between “Satisfaction” and “Impact on Daily Life” is significantly influenced by prior treatment experiences and the quality of doctor-patient interactions. Patients with positive past experiences approach new interventions with optimism, believing in the potential for their daily lives to be improved [44]. Conversely, individuals with unsatisfactory treatments may have lower expectations and reduced satisfaction. The quality of doctor-patient interaction plays a vital role in shaping this connection. Positive, empathetic rapport instills trust and confidence, enhancing treatment expectations and overall satisfaction.

In the context of KOA management, patients often express concerns about hands-on techniques like massage and manual therapy. These concerns revolve around the nature of these treatments aligning with their comfort levels and expectations. Addressing these specific concerns is crucial to ensuring that these therapies are gentle, soothing, and aligned with patients’ individual preferences and needs.

The concept of the doctor-patient interaction cycle introduces nuanced subjectivity in patients, particularly during trial implementation. Despite standardization efforts, patients exhibit diverse and sometimes profound subjective experiences, shaping treatment perspectives uniquely. This subjectivity extends to patient communication during sessions, unraveling the complexities of their ailments. The transformative influence of this verbal exchange, often overlooked, presents uncharted terrain for investigation, suggesting potential psychosomatic dimensions in manual therapy interventions.

Under the Biopsychosocial (BPS) medical model, disease management involves not only biological treatments but also the combined influence of psychological and social factors. In manual therapy, doctor-patient interaction is not merely a process of information exchange but also a key determinant of patient compliance, subjective experience, and clinical outcomes. Patients’ pain perception, trust in treatment, and proactive engagement in disease management are influenced by the physician’s words, attitude, and behavior. Therefore, optimizing doctor-patient interaction while addressing patients’ psychological needs and social support can significantly enhance treatment outcomes. In modern medical research, the use of language has a profound impact on treatment outcomes. Positive suggestions from doctors (such as emphasizing the effectiveness of treatment and reducing anxiety) can enhance patients’ confidence, improve their treatment adherence, and potentially trigger a placebo effect that alleviates symptoms. Conversely, negative suggestions (such as emphasizing the irreversibility of pain or the severity of the condition) may lead to a nocebo effect, increasing patients’ discomfort and anxiety. Therefore, in manual therapy, physicians should consciously use positive language to shape patients’ optimistic perceptions of treatment, maximizing its psychological benefits.

### Treatment goals and outcomes

“Treatment Goals and Outcomes” involves an in-depth analysis of the two central facets in KOA management: pain alleviation and the restoration of athletic capabilities [45]. These objectives form the core focus of therapeutic interventions in the care of KOA patients. The goal of alleviating pain symptoms is illuminated as a paramount objective, aiming not only to mitigate discomfort but also to significantly enhance patients’ overall quality of life. Simultaneously, the aspiration to restore athletic capabilities is highlighted, emphasizing the importance of interventions that go beyond mere symptom management, seeking to empower patients by improving their physical function and mobility. This dual-focus approach reflects a holistic understanding of KOA, recognizing that effective care extends beyond immediate symptom relief to encompass broader outcomes related to patients’ daily activities and well-being. While these objectives are universally recognized in clinical practice, the ways in which patients prioritize and perceive these goals can vary significantly. This variability is influenced by a range of factors, including age, gender, occupational and social roles, previous injury history, symptom severity, and comorbidities. A deeper understanding of these diverse perspectives is essential to tailoring treatment approaches that align with individual patient needs and expectations.

As we explore the ramifications of our research, it becomes clear that the crux of successful KOA management lies in adopting a patient-centered approach [46]. The analysis has brought to light the varied experiences and expectations of those contending with KOA, highlighting the imperative for a nuanced and tailored approach in clinical practice. In this framework, accentuating the pivotal role of doctor-patient communication, adaptable treatment approaches, and the integration of healthcare services.

The heterogeneity in patient perspectives presents both a challenge and an opportunity for KOA management. Clinicians must navigate these diverse expectations by engaging in shared decision-making and aligning treatment goals with patient priorities. A one-size-fits-all approach is unlikely to meet the needs of all KOA patients. Instead, a more individualized strategy that incorporates patient education, goal-setting discussions, and adaptable treatment plans can enhance treatment adherence and satisfaction. Furthermore, qualitative research in this area should continue to explore how demographic, occupational, and psychosocial factors shape treatment expectations, allowing for the development of more inclusive and personalized care models.

### Implications

The “Information Gap” theme underscores the imperative for a patient-centric approach to information dissemination. It emphasizes the critical need for clear, accessible, and patient-friendly resources. This finding highlights the vital role of healthcare providers and educators in addressing the information gap. In essence, this interpretation urges exploration into the lived experiences and challenges faced by individuals with limited understanding on this journey. Furthermore, the importance of patient education and guidance is accentuated, particularly within the context of the doctor’s treatment services and overall health management. Providing essential information pathways and teaching patients how to access relevant information is crucial for effective healthcare. Healthcare professionals should prioritize equipping patients with the necessary knowledge and resources, empowering them to manage their KOA condition effectively.

Addressing the multifaceted role of exercise in KOA necessitates consideration of fitness levels, pain thresholds, and specific limitations [47–49]. Therefore, the development of personalized exercise plans for patients is crucial. Patient education must emphasize proper exercise, providing reliable resources for guidance. Continuous monitoring allows healthcare providers to optimize outcomes and engage patients in decision-making for their exercise plans. Implementing effective pain management strategies ensures that discomfort doesn’t hinder adherence to exercise regimens. This approach seeks to balance using exercise as therapy while minimizing risks associated with improper exercise in the context of KOA.

Healthcare providers must consider not only clinical aspects but also the holistic impact on patients’ daily lives, expectations, and satisfaction. Effective doctor-patient communication and addressing procedural concerns are crucial, ensuring well-received treatment plans aligned with patients’ lifestyles and improving overall satisfaction and outcomes. Understanding these dynamics is paramount for healthcare providers. Recognizing the influence of prior experiences and fostering effective doctor-patient interactions allows tailoring treatments not only for clinical symptoms but also to enhance patients’ daily lives. This holistic approach is essential for improving overall satisfaction and the quality of life for individuals with KOA.

In summary, the crux of successful KOA management lies in adopting a patient-centric approach, acknowledging the varied experiences and expectations of those contending with this condition. By accentuating doctor-patient communication, adaptable treatment approaches, and the integration of healthcare services, our study contributes to a comprehensive model for effective KOA management. This model recognizes the imperative to address the information gap, personalize exercise plans, and enhance overall patient satisfaction, thus paving the way for a more holistic and tailored approach in clinical practice.

### Strengths of this study

This study utilizes a qualitative research design to explore various influencing factors in manual therapy for KOA, generates a doctor-patient interaction model for manual therapy in KOA, and aims to identify key priorities for clinicians in optimizing treatment strategies and delivering holistic care.

It employs a patient-centric approach, capturing diverse patient experiences and perceptions, including potential doctor-patient interactions, for application in medical practice.

It provides an in-depth exploration of patient experiences with manual therapy interventions (e.g., Tuina and manual physical therapy), highlighting both perceived benefits (e.g., pain relief) and limitations (e.g., variability in treatment responses).

### Limitations of this study

The sociocultural and clinical context of outpatient care in China (e.g., doctor-patient relationships, integration of traditional medicine) may limit the transferability of results to populations in other healthcare systems or cultural environments.

This study focuses solely on Tuina and manual physical therapy, which may not fully capture patient experiences with other treatment modalities (e.g., medication or surgery).

As a nested qualitative trial, this research inherits limitations from the parent trial, such as standardized intervention protocols that restrict exploration of individualized treatment variations. This may affect the flexibility of applying findings to diverse clinical practices.

## Conclusion

Our study generated three themes—Understanding and Impact, Treatment Expectations and Satisfaction, and Treatment Goals and Outcomes, and developed a manual therapy model based on these themes. This model highlights the dynamic interplay of socio-cultural, clinical, and interpersonal factors in shaping patients’ experiences and perceptions, particularly the persistent “Information Gap” exacerbated by hierarchical doctor-patient dynamics in China. Patients’ divergent priorities—younger individuals seeking functional recovery for high-intensity activities versus older adults prioritizing daily independence—call for precision care model that integrate personalized goal-setting, real-time feedback, and psychological support. While manual therapies like Tuina show promise, future research should expand to multidisciplinary models and cross-cultural validation, ensuring that patient-centered communication and adaptive strategies bridge the gap between standardized protocols and individualized needs.

## Electronic supplementary material

Below is the link to the electronic supplementary material.

Supplementary Material 1

## Data Availability

No datasets were generated or analysed during the current study.
