# Supplementary material for: Experiences and perceptions of Chinese patients enrolled in a clinical trial assessing tuina and manual therapies for knee osteoarthritis: a nested qualitative study
Source: BMC Complement Med Ther. 2025 May 28;25:191. doi: 10.1186/s12906-025-04926-7 (PMC12121067; doi:10.1186/s12906-025-04926-7)
Supplement: Supplementary file 1 — Supplementary Material 1 [file 12906_2025_4926_MOESM1_ESM.docx]

| Author | Credentials | Occupation | Gender | Experience in qualitative research | Clinical experience | Participate in clinical intervention | Participate in interview | Transcribed into NVivo | Data Analysis |
| --- | --- | --- | --- | --- | --- | --- | --- | --- | --- |
| Luping Liu | MD | Student | Male | 2 years | 3 years | No |  |  | Yes |
| Lingyun Zhang | Bachelor of Medicine | Student | Female | 2 years | 2 years | No |  |  | Yes |
| Sina Li | MD | Student | Female | 3 years | 4 years | No |  |  |  |
| Meiling Cai | MD | Student | Female | 3 years | 4 years | No | Yes | Yes |  |
| Siyu Han | MD | Student | Female | 3 years | 4 years | No | Yes | Yes | Yes |
| Zhiwen Weng | MD | Nurse | Female |  |  | Yes |  |  |  |
| Qianji Chen | MD | Student | Female | 3 years | 4 years | No |  |  |  |
| Yixuan Gao | Bachelor of Medicine | Student | Female | 2 years | 2 years | No |  |  |  |
| Xiaoming Yang | MD | Doctor | Male |  | 12 years | Yes |  |  |  |
| Yang Zhang | MD | Doctor | Male |  | 12 years | Yes |  |  |  |
| Duoduo Li | MD | Doctor | Male | 7 years | 15years | Yes |  |  |  |
| Changxin Liu | MD,PhD | Doctor | Male |  | 35 years | Yes |  |  |  |
| Ya’nan Sun | MD | Doctor | Female | 5 years |  | Yes |  |  |  |
| Xiyou Wang | MD | Doctor | Male | 5 years | 27 years | Yes |  |  |  |
| Changhe Yu | MD,PhD | Doctor | Male | 9 years | 12 years | Yes |  |  | Yes |
